# Risk factors for low adherence to methylphenidate treatment in pediatric patients with attention-deficit/hyperactivity disorder

**DOI:** 10.1038/s41598-021-81416-z

**Published:** 2021-01-18

**Authors:** Asami Ishizuya, Minori Enomoto, Hisateru Tachimori, Hidehiko Takahashi, Genichi Sugihara, Shingo Kitamura, Kazuo Mishima

**Affiliations:** 1 Department of Psychiatry and Behavioral Neurosciences, Graduate School of Medical and Dental Sciences, Tokyo Medical and Dental University, Bunkyo-ku, Tokyo, Japan; 2 Department of Neuropsychiatry, Akita University Graduate School of Medicine, Hondo 1-1-1, Akita-city, Akita 010-8543 Japan; 3 Department of Sleep-Wake Disorders, National Center of Neurology and Psychiatry, National Institute of Mental Health, Kodaira, Tokyo, Japan; 4 Department of Medical Technology, School of Health Sciences, Tokyo University of Technology, Tokyo, Japan; 5 International Institute for Integrative Sleep Medicine, University of Tsukuba, Tsukuba, Japan; 6 Department of Clinical Epidemiology, Translational Medical Center, National Center of Neurology and Psychiatry, Kodaira, Tokyo, Japan

**Keywords:** Medical research, Risk factors, Neurodevelopmental disorders, ADHD

## Abstract

Poor adherence is a major concern in the treatment of attention-deficit/hyperactivity disorder (ADHD). The objective of this study was to evaluate factors linked to early interruption of and low adherence to treatment with osmotic-release oral system methylphenidate hydrochloride (OROS-MPH) in pediatric patients with ADHD. A total of 1353 young people (age 6–17 years) with a diagnosis of ADHD who newly started OROS-MPH were extracted from the pharmacoepidemiological data of 3 million people in Japan. The cohort was retrospectively surveyed every month for 12 months. Ten possible risk factors were extracted from the data and analyzed by multivariable logistic regression. Sensitivity analysis was conducted to ensure the robustness of the analysis. The results revealed that treatment adherence was generally poor, with a tendency for discontinuation in the early stage. Multivariable logistic regression results showed that adherence is reduced by female sex, lower starting dose, and concomitant atomoxetine or hypnotics. These findings may help clinicians to predict the risk of poor adherence in the early stage of treatment and improve not only patients’ symptoms, but also their quality of life.

## Introduction

Attention-deficit/hyperactivity disorder (ADHD) is a common chronic neurodevelopmental disorder characterized by symptoms of inattention, hyperactivity, and impulsivity. Individuals with ADHD are at high risk of comorbid psychiatric disorders, such as oppositional defiant disorder, conduct disorders, depression, bipolar disorders, and anxiety disorders^1^. The onset of ADHD occurs mostly in childhood, but 50% to 80% of diagnosed children continue to demonstrate ADHD symptoms into adolescence or adulthood^2–5^. The symptoms of ADHD and comorbid disorders can impair an individual’s ability to function in social circumstances, resulting in academic, familial, and social problems^6,7^.

In the latest Japanese guidelines for the diagnosis and treatment of ADHD, released in 2016^8^, the management of children with ADHD should begin with psychosocial treatment. Additional administration of pharmacological therapy should be considered when psychosocial treatments are insufficient. However, as shown by fact-finding surveys on pharmacotherapy for ADHD, most physicians support the need for pharmacotherapy^9^.

Four types of ADHD medication are currently available in Japan: osmotic-release oral system methylphenidate hydrochloride (OROS-MPH, CONCERTA), atomoxetine (ATX, STRATTERA), guanfacine hydrochloride (INTUNIV), and lisdexamfetamine dimesylate (VYVANSE). Of these medications, OROS-MPH is the most prescribed. Due to its addictive profile, the circulation management committee strictly controlled its prescription by limiting prescribers’ certification and the prescription duration until the ADHD proper distribution management system is established in December 2019. Accordingly, there is a high likelihood of proper prescription of OROS-MPH in Japan.

The latest Japanese guidelines^8^ recommended that termination of medication be considered when patients are stable for more than 1 year after they obtain a score of 61 or above on the Global Assessment of Functioning (GAF) scale. Therefore, patients need to adhere to the prescribed treatment regimens for at least 1 year.

Despite the high reliability of proper prescription of OROS-MPH medication in Japan because of its strict control, there is an issue with patients’ unstable patterns of outpatient visits and oral intake in clinical practice. This leads to poor adherence or discontinuation of the treatment in the early stage, which could limit treatment effects and increase the risk of comorbid psychiatric disorders. There is a considerable need to understand the prescription patterns of ADHD medications and to identify risk factors associated with poor adherence in order to help patients to adhere to their recommended treatment regimen and to facilitate proper intervention. However, to our knowledge, no study has examined the actual prescription patterns of OROS-MPH and analyzed the possible factors associated with poor adherence in Japan. Therefore, in this study, we evaluated the current prescription trend of OROS-MPH in pediatric ADHD patients in Japan. We also analyzed the factors that could predict poor adherence in the early stage of the treatment, based on data from the claims database.

## Methods

All procedures were performed in accordance with the ethical guidelines for epidemiological research issued by the Japanese Ministry of Health, Labour and Welfare. This study was approved by the Ethics Committee of Akita University Graduate School of Medicine, Akita, Japan, which waived the requirement for obtaining written informed consent.

### Data source

The data used in this research were provided by JMDC Inc. (Tokyo, Japan). JMDC Inc. has been collecting claims information from occupation-based health insurance agencies for corporate employees and their dependents since 2005^10^. All health insurance data included in the JMDC database are anonymized, and permission for secondary use of the data has been obtained from the subscriber.

The number of individuals registered in JMDC is about 3 million, which is approximately 2.5% of the entire Japanese population as of June 2016. Each record includes an encrypted personal identifier, age, sex, diagnoses, and prescriptions. The diagnoses are based on International Statistical Classification of Diseases and Related Health Problems, 10th revision (ICD-10) diagnostic codes. The prescription information includes WHO Anatomical Therapeutic Chemical (WHO-ATC) classification system codes, drug name, days of supply, dosage information, and mode of prescription. The date of service is specified up to the month and year.

### Study population

We selected outpatients who were 6 to 17 years when they were first prescribed OROS-MPH between December 2007 and May 2015. We selected only new users who had not been prescribed MPH within 1 year prior to their first prescription during our observation period. Then, we extracted individuals who could be observed for at least 1 year. We also excluded individuals who had a diagnosis of narcolepsy because they are prescribed methylphenidate hydrochloride and not OROS-MPH. A total of 1353 children were included in this study (Fig. 3).

### Study variables

#### Patient persistence and adherence

Medication compliance has two indicators: “persistence” and “adherence”. Persistence is usually calculated by the period until the discontinuation of the initial treatment. In this study, the discontinuation of the medication was defined as the beginning of a gap of more than 3 months in prescriptions for OROS-MPH. We named this gap period GAP3M in this study. On the other hand, adherence was assessed using the MPR, which reflects the proportion of months that patients were in possession of the medication within a specific period. We calculated the MPR by summing the number of months in which patients received their prescription and dividing it by the follow-up period in months (12 months). The conventional 50% cutoff was used.

#### Risk factors

We selected the possible risk factors that might interfere with medication adherence. As mentioned in previous studies, many factors are related to medication continuation and adherence. These include specific child and adolescent characteristics, such as age^11–16^, male sex^11^, and ethnic background^13^, clinical characteristics, such as symptom severity^13,14,16^ and amount of symptom reduction^17,18^, quality of ADHD care, such as medication doses^15,17,18^, adverse events^19^, and concomitant medication^11^, and factors related to parents or families, such as belief in treatment^12^, poor family support^20^, and family history of ADHD^14^. In this study, we selected sex, age at first OROS-MPH prescription, clinician specialties, mean daily dosage prescribed in the first 3 months, and concomitant medication in the first 3 months.

Patient were divided into three age groups as follows: 6–12 years of age (elementary school students), 13–15 years of age (junior high school students), and 16–17 years of age (high school students or junior high school graduates) according to the Japanese school system.

The mean daily dosage prescribed in the first 3 months was calculated by dividing the sum of the total prescription in the first 3 months by 90 days. The results were divided into three groups: a low-dose group (< 18 mg), middle-dose group (18–27 mg), and high-dose group (≥ 27 mg) according to the dosing schedule based on approvals granted by the Japanese regulatory authority^21^. Clinician specialties were divided into three groups as follows: psychiatry/psychosomatic medicine, pediatrics, and others. The reasoning for this division of departments is that only members of the Japan Pediatric Society or the Japanese Society of Psychiatry are under the control of the circulation management committee.

The types of concomitant medications were divided into six groups: ATX, antipsychotics, hypnotics, antidepressants, antiepileptics, and anxiolytics (see Supplementary Table S2 online).

### Statistical analysis

The estimated prescription rate of OROS-MPH was calculated, as well as the actual prescription rate in the target group, which was the population who received the prescription from December 2007 to April 2016. The estimated prescription rate for the whole population (from 6 to 17 years) was calculated by correction for the population statistics issued by the statistics bureau of Japan^22^. Kaplan–Meier survival curves were used to estimate the continuing prescription of OROS-MPH.

We used multivariable logistic regression analysis to assess the association between risk factors and poor adherence. We identified ten possible risk factors (Table 1) and divided patients into two groups: poor adherence (MPR below 0.5) and good adherence (MPR above 0.5). The logistic regression analysis was conducted using IBM SPSS Statistics version 22 (IBM, Armonk, NY, USA). The analyses were two-tailed, and *P* values < 0.05 were considered statistically significant.

**Table 1:** Demographic characteristics of the participants.

	N (%)	MPR (Mean, SD)	MPR < 0.5 (N, %)	MPR ≥ 0.5 (N, %)
Total	1353 (100%)	0.51 (± 0.32)	591 (43.7%)	762 (56.3%)
**Sex**				
Male	1141 (84.3%)	0.53 (± 0.32)	469 (41.1%)	672 (58.9%)
Female	212 (15.7%)	0.43 (± 0.33)	122 (57.5%)	90 (42.5%)
*Age at their first OROS-MPH prescription*				
**Mean, SD 9.65 (± 2.85), Median 9**				
6–12 years old	1084 (80.1%)	0.52 (± 0.32)	460 (42.4%)	624 (57.6%)
13–15 years old	225 (16.6%)	0.50 (± 0.32)	106 (47.1%)	119 (52.9%)
16–17 years old	44 (3.3%)	0.43 (± 0.33)	25 (56.8%)	19 (43.2%)
**Clinician specialties**				
Psychiatry and Psychosomatic Medicine	45 (3.3%)	0.45 (± 0.36)	25 (55.6%)	20 (44.4%)
Pediatrics	151 (11.2%)	0.54 (± 0.32)	57 (37.7%)	94 (62.3%)
Others	1,157 (85.5%)	0.51 (± 0.32)	509 (55.2%)	648 (44.8%)
**Mean daily dosage prescribed in the first 3 months**				
Low (< 18 mg)	653 (48.3%)	0.43 (± 0.32)	348 (53.5%)	305 (46.7%)
Middle (18–27 mg)	515 (38.1%)	0.58 (± 0.32)	183 (35.5%)	332 (64.5%)
High (≥ 27 mg)	185 (13.7%)	0.61 (± 0.29)	60 (32.4%)	125 (67.6%)
*Concomitant use of other medications in the first 3 months*				
**Atomoxetine**				
Non-combination group	1139 (84.2%)	0.54 (± 0.32)	454 (39.9%)	685 (60.1%)
Combination group	214 (15.8%)	0.38 (± 0.34)	137 (64.0%)	77 (36.0%)
**Antipsychotic**				
Non-combination group	1149 (84.9%)	0.52 (± 0.32)	485 (42.2%)	664 (57.8%)
Combination group	204 (15.1%)	0.47 (± 0.35)	106 (52.0%)	98 (48.0%)
**Hypnotic**				
Non-combination group	1296 (95.8%)	0.52 (± 0.32)	554 (42.7%)	742 (57.3%)
Combination group	57 (4.2%)	0.41 (± 0.35)	37 (64.9%)	20 (35.1%)
**Antidepressant**				
Non-combination group	1322 (97.7%)	0.51 (± 0.32)	578 (43.7%)	744 (56.3%)
Combination group	31 (2.3%)	0.55 (± 0.36)	13 (41.9%)	18 (58.1%)
**Antiepileptic**				
Non-combination group	1343 (99.3%)	0.51 (± 0.32)	588 (43.8%)	755 (56.2%)
Combination group	10 (0.7%)	0.63 (± 0.37)	3 (30.0%)	7 (70.0%)
**Anxiolytic**				
Non-combination group	1352 (99.9%)	0.51 (± 0.32)	590 (43.6%)	762 (56.4%)
Combination group	1 (0.1%)	0.08	0 (0.0%)	1 (100.0%)

MPR, medication possession ratio; N, number; OROS-MPH, osmotic-release oral system methylphenidate hydrochloride; SD, standard deviation.

We also conducted sensitivity analysis to ensure the robustness of the analysis. We identified two factors from the literature: ADHD severity and adverse events. ADHD severity was thought to interfere with the mean daily dosage prescribed in the first 3 months, whereas adverse events are thought to interfere with concomitant hypnotic use. The OR for reduced adherence and the ratio of patients who might have possible confounding factors were determined from the literature^16,19,23,24^. Sensitivity analysis was performed using the method provided by Fox et al.^25^.

## Results

### Estimated prescription rate

**Figure 1:**
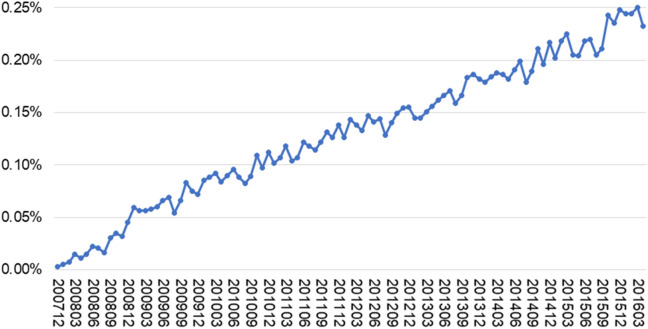
Prevalence estimates of OROS-MPH in the Japanese population aged 6–17 years. Prevalence estimates were corrected with population statistics issued by the statistics bureau of Japan (27). OROS-MPH, osmotic-release oral system methylphenidate hydrochloride.

During the period from the launch of OROS-MPH in Japan (December 2007) to the last month of data acquisition from the Japan Medical Data Center (JMDC; April 2016), the estimated prescription rate gradually increased from 0.003 to 0.23% (Fig. 1).

### Prescription patterns

**Figure 2:**
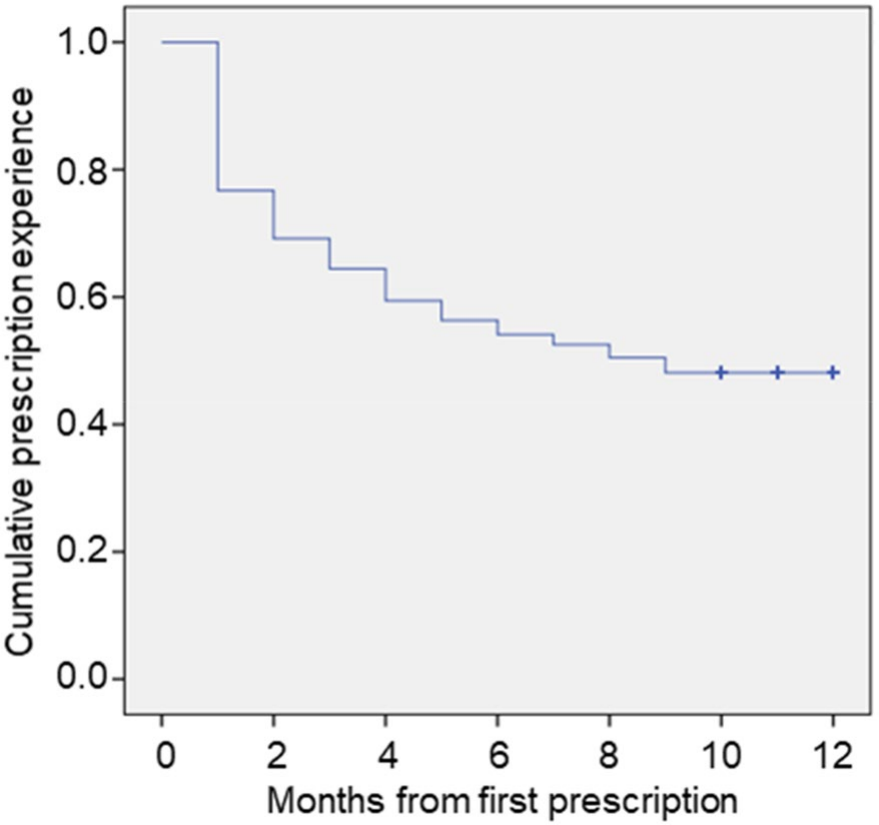
Kaplan–Meier survival curve for prescription continuance.

**Figure 3:**
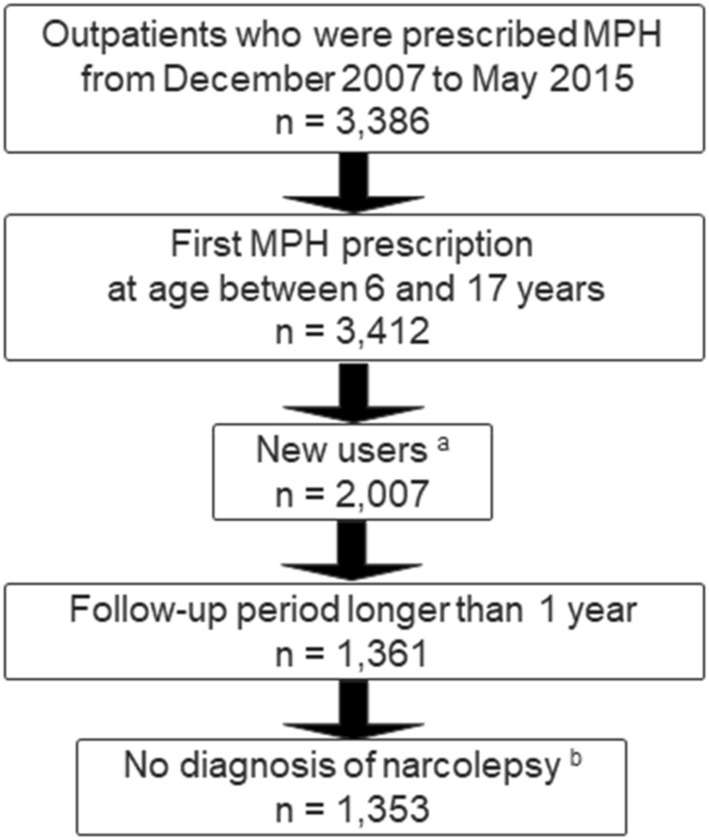
Participants’ flow diagram. ^a^New users: patients who had not been prescribed MPH within 1 year prior to their first prescription month during our observation period. ^b^No diagnosis of narcolepsy: patients who were prescribed OROS-MPH were chosen. MPH, methylphenidate hydrochloride; OROS-MPH, osmotic-release oral system methylphenidate hydrochloride.

This study included individuals who first started OROS-MPH medication for ADHD between December 2007 and May 2015. Table 1 shows the characteristics of the participants, which are summarized using descriptive statistics (mean, standard deviation, percentage). Prescription durations are plotted as a Kaplan–Meier survival curve in Fig. 2. The prescription patterns varied and the mean medication possession ratio (MPR) was 0.51 ± 0.32 (Table 1), despite the strict control of the circulation management committee. In our study, only 8.6% of patients (116 of 1353) had no gap period at all, whereas 51.9% (702 of 1353) had a gap period of more than 3 months. Of those with a gap less than 2 months (535 patients), 95.7% (512 patients) resumed the medication and 99.4% (532 patients) had an MPR exceeding 0.5. In contrast, of those with a gap more than 3 months (702 patients), only 17.9% (125 patients) resumed the medication and 16.2% (114 patients) had an MPR exceeding 0.5. Moreover, 90.8% of the patients with an MPR greater than 0.5 (692 of 762) continued to take the medication until the end of the follow-up period. Based on these results, we defined an MPR greater than 0.5 as an indicator of adherence and a gap of more than 3 months (GAP3M) as an indicator of discontinuation (Fig. 3).

### Multivariable logistic regression

The results of the multivariable logistic regression are shown in Table 2. For patient characteristics, female patients were more likely to show poor adherence than male patients (odds ratio [OR], 0.565; 95% confidence interval [CI], 0.414–0.773; *P* = 0.000). The mean MPR became smaller as the age at first prescription increased, but there was no statistically significant effect of age on the mean MPR. In addition, clinician specialties were not found to be associated with adherence.

**Table 2:** Risk factors related to adherence during the follow-up period among new users of OROS-MPH (n = 1353).

	OR	95% CI to *P* value	*P* value
**Sex**			
Male		Ref	Ref
Female	0.5650	**0.414–0.773**	0.0000
**Age at first OROS-MPH prescription**			
6–12 years old	Ref	Ref	Ref
13–15 years old	0.7980	0.583–1.093	0.1600
16–17 years old	0.5420	0.275–1.069	0.0770
**Clinical departments**			
Psychiatry and Psychosomatic Medicine	Ref	Ref	Ref
Pediatrics	1.8610	0.915–3.785	0.0860
Others	1.4380	0.765–2.706	0.2600
**Mean daily dosage prescribed in the first 3 months**			
Low (< 18 mg)	Ref	Ref	Ref
Middle (18–27 mg)	1.9860	**1.554–2.536**	0.0000
High (≥ 27 mg)	2.4990	**1.731–3.608**	0.0000
*Concomitant use of other medications in the first 3 months*			
**Atomoxetine**			
Yes	0.3760	**0.273–0.517**	0.0000
No	Ref	Ref	Ref
**Antipsychotic**			
Yes	0.7720	0.559–1.066	0.1160
No	Ref	Ref	Ref
**Hypnotic**			
Yes	0.5360	**0.291–0.985**	0.0450
No	Ref	Ref	Ref
**Antidepressants**			
Yes	1.3710	0.551–3.411	0.4980
No	Ref	Ref	Ref
**Antiepileptic**			
Yes	4.0850	0.644–25.906	0.1350
No	Ref	Ref	Ref
**Anxiolytic**			
Yes	0.0000	0.0000	1.0000
No	Ref	Ref	Ref

*P* values in bold indicate a significant difference from the reference group at the α = 0.05 level.

CI, confidence interval; MPR, medication possession ratio; OR, odds ratio; OROS-MPH, osmotic-release oral system methylphenidate hydrochloride; Ref, reference.

Regarding the mean daily dosage prescribed in the first 3 months, adherence was likely to be poor if patients were prescribed doses lower than 18 mg per day and was significantly worse than if they were prescribed 18–27 mg per day and ≥ 27 mg per day (OR, 1.986; 95% CI, 1.554–2.536; *P* = 0.000; OR, 2.499; 95% CI, 1.731–3.608; *P* = 0.000, respectively).

In terms of the types of concomitant medication in the first 3 months, concomitant use of ATX (OR, 0.376; 95% CI, 0.273–0.517; *P* = 0.000) or hypnotics (OR, 0.536; 95% CI, 0.291–0.985; *P* = 0.045) was significantly associated with poor adherence.

### Sensitivity analysis

The results of the sensitivity analysis are shown in Supplementary Table S1 online. Considering the calculated original risk ratio of 1.54, ADHD severity was not considered to have a significant effect on the relationship between adherence and the mean daily dosage, even in the most plausible pattern (1.6 for the estimated risk ratio of the confounding factor, about 60% for the proportion of patients who use OROS-MPH more than 18 mg per day and about 50% for the proportion of patients who use OROS-MPH less than 18 mg per day). Therefore, the severity of ADHD was not considered to interfere with the results.

Regarding the effect of adverse events on the relationship between adherence and concomitant hypnotic use, the adjusted risk ratio was 1.46 in the most plausible pattern (1.5 for the estimated risk ratio of the confounding factor, 70% for the proportion of the patients in the concomitant hypnotic use group, and 60% in the non-concomitant group). Considering the original risk ratio of 1.52, it is necessary to be cautious about the risk of the occurrence of adverse events in relation to adherence and the concomitant use of hypnotics, and further consideration of this aspect is needed.

## Discussion

First, this study revealed the actual status of OROS-MPH prescription. The trend for a gradual increase in prescription rates is similar to that of other countries^26–29^. However, the prevalence of ADHD drug use in pediatric patients was lower in Japan (0.4%)^30^ than in Norway and the United States (1.4–5.3%)^31,32^ but similar to that in Italy, France, and the UK (0.2–0.5%)^33–35^. In addition, the share of OROS-MPH in 2014 is lower in Japan (64%)^30^ than in Germany, the UK, and Norway (75–100%)^32,33,36^. Thus, both the prescription of ADHD drug and the share of OROS-MPH are low in Japan.

One of the reasons for this situation in Japan could be the strict control of OROS-MPH prescription by the circulation management committee to limit the qualification of prescribers and the prescription period. Another possible reason is the indication for the first-line drug of ADHD medication in the Japanese guidelines^8^. Both OROS-MPH and ATX are equally listed as first-line drugs, in contrast to other guidelines that recommend psychostimulants as first choice^37–39^. Although there are differences in efficacy and tolerability among ADHD medications^40,41^, Japanese physicians may prefer ATX for the initial drug because it has no restriction.

Second, we revealed the erratic prescription patterns of OROS-MPH and analyzed the possible factors that could predict the risk of low adherence in the early stage of the OROS-MPH prescription. This erratic pattern could be caused by patients forgetting to take the medication, intentional discontinuation, or “drug holidays”. The term “drug holiday” refers to the deliberate interruption of pharmacotherapy for a defined period and for a specific clinical purpose^42^. Although guidelines recommend patients take “drug holidays” when the use is extended over a long period^8,39^, some patients take these holidays on weekends or during school holidays from the early stage of the treatment.

The mean MPR in this study was 0.51 ± 0.32. This is similar to the mean MPR of 0.52 ± 0.30 for extended-release stimulants in Texas^11^ but less than the mean MPR of 0.64 found in the Netherlands^43^. Common adherence thresholds in previous studies were 0.8^11,43,44^ or 0.7^45^. However, as shown in our results, 90.8% of those with an MPR greater than 0.5 continued to take their medication until the end of the follow-up period. Therefore, we defined an MPR greater than 0.5 as an indication of adherence.

Among possible risk factors for low adherence detected in our study, sex differences had a statistically significant influence on adherence. Compared with girls, boys with ADHD have a tendency to show more externalized symptoms^46^. Although OROS-MPH can reduce both externalized and internalized symptoms^19^, improvements in externalized symptoms are more readily noticed by caregivers. Therefore, boys may more easily perceive the effects of the medication and thus be more likely to continue to take it, as shown in our results.

Regarding a mean daily dosage lower than 18 mg per day, an insufficient treatment effect due to an insufficient dose might be the reason for lower adherence^47^. Although the occurrence of adverse events might affect adherence, the adverse events that occur in the short term are mostly non-serious^48,49^ and lead to withdrawal in less than 10% of the patients who experience them^49^. In addition, lower doses are usually less likely to cause adverse events compared with higher doses.

In terms of concomitant medication, only ATX and hypnotics were linked to adherence. The common reasons for the prescription of concomitant ADHD medications are an inadequate response and intolerance to the previous treatment^50^. Therefore, the patients who were prescribed ATX in the early stage could be in the process of treatment switching or augmentation. Patients undergoing switching or augmentation are likely to have lower adherence because of the risk of additive adverse effects caused by the combination use. Therefore, it is understandable that the patients with concomitant ATX use had lower adherence.

Patients with concomitant psychotropic use had lower MPRs in our study. However, only hypnotics exhibited a statistically significant association with adherence. Sleep disturbances can appear as ADHD symptoms^51–53^. Therefore, patients with concomitant hypnotic use in the early stage are presumed to originally have had moderate or severe sleep problems. OROS-MPH may worsen sleep status as an adverse effect^54,55^, which may negatively affect adherence.

There are limitations to the use of the JMDC dataset. It is one of the largest claims databases in Japan but includes only 1–2% of all Japanese inhabitants. Additionally, all participants were recipients (or the family members of recipients) of employee’s health insurance (*Kenkō-Hoken*). Therefore, there is a potential for bias in socioeconomic and family backgrounds, which could affect adherence^12,14,20^. In addition, there is a lack of clinical detail in the database, such as ADHD symptoms and severity, incidence of adverse events, and coexisting psychiatric diseases. Therefore, it was not possible to determine whether adherence was due to remission of ADHD, ineffectiveness of medication, or aggravated coexisting psychiatric diseases. Thus, we evaluated the effect of these factors by conducting sensitivity analysis. Another limitation is that this database represents the prescription records and not the actual oral intake records. Thus, there is a possibility that patients did not take the medication, even with the prescription. These limitations should be considered in future studies.

Despite these limitations, the findings of our study reveal the current state of pharmacotherapy for ADHD using OROS-MPH in Japan and provide useful insights into its resolution. Due to the increased prevalence of ADHD and use of psychostimulants, the inappropriate use of and low adherence to psychostimulants have been major problems in Japan. Appropriate medications for young ADHD patients are important not only to improve ADHD symptoms, but also to prevent the resultant disabilities and difficulties in their daily lives.

This is the first study to provide clinically important evidence of the relationship between adherence and the possible risk factors in OROS-MPH treatment for children and adolescents with ADHD in Japan using a large-scale claims database. These results can help clinicians to predict the risk of poor adherence in the early stage of treatment and improve not only patients’ symptoms, but also their quality of life.

## Supplementary Information

Supplementary Information.

## Data Availability

The data that support the findings of this study are available from JMDC but restrictions apply to the availability of these data. The data were used under license for the current study and thus are not publicly available. However, the data are available from the authors upon reasonable request and with permission of JMDC.

## References

[1] Diagnostic and Statistical Manual of Mental Disorders, 5th edition (DSM-V) (American Psychiatric Association, Washington, DC, 2013).

[2] Barbaresi WJ (2013). Mortality, ADHD, and psychosocial adversity in adults with childhood ADHD: a prospective study. Pediatrics.

[3] Biederman J, Petty CR, Clarke A, Lomedico A, Faraone SV (2011). Predictors of persistent ADHD: an 11-year follow-up study. J. Psychiatr. Res..

[4] Cheung CHM (2015). Childhood predictors of adolescent and young adult outcome in ADHD. J. Psychiatr. Res..

[5] Lara C (2009). Childhood predictors of adult attention-deficit/hyperactivity disorder: results from the World Health Organization World Mental Health Survey Initiative. Biol. Psychiatry.

[6] Harpin VA (2005). The effect of ADHD on the life of an individual, their family, and community from preschool to adult life. Arch. Dis. Child..

[7] Hoza B (2005). What aspects of peer relationships are impaired in children with attention-deficit/hyperactivity disorder?. J. Consult. Clin. Psychol..

[8] Saito M (2016). Japanese Guideline for Diagnosis and Treatment of AD/HD.

[9] Miyachi T (2010). The investigation into the actual conditions of medication for the child with attention deficit/hyperactivity disorder (AD/HD) in Japan. Psychiatr. Neurolo. Paediatr. Jpn..

[10] Kimura S, Sato T, Ikeda S, Noda M, Nakayama T (2010). Development of a database of health insurance claims: standardization of disease classifications and anonymous record linkage. J. Epidemiol..

[11] Barner JC, Khoza S, Oladapo A (2011). ADHD medication use, adherence, persistence and cost among Texas Medicaid children. Curr. Med. Res. Opin..

[12] Brinkman WB, Sucharew H, Majcher JH, Epstein JN (2018). Predictors of medication continuity in children with ADHD. Pediatrics.

[13] Faraone SV, Biederman J, Zimmerman B (2007). An analysis of patient adherence to treatment during a 1-year, open-label study of OROS methylphenidate in children with ADHD. J. Atten. Disord..

[14] Gau SS-F (2008). National survey of adherence, efficacy, and side effects of methylphenidate in children with attention-deficit/hyperactivity disorder in Taiwan. J. Clin. Psychiatry.

[15] Sanchez RJ, Crismon ML, Barner JC, Bettinger T, Wilson JP (2005). Assessment of adherence measures with different stimulants among children and adolescents. Pharmacotherapy.

[16] Thiruchelvam D, Charach A, Schachar RJ (2001). Moderators and mediators of long-term adherence to stimulant treatment in children with ADHD. J. Am. Acad. Child Adolesc. Psychiatry.

[17] Marcus SC, Wan GJ, Kemner JE, Olfson M (2005). Continuity of methylphenidate treatment for attention-deficit/hyperactivity disorder. Arch. Pediatr. Adolesc. Med..

[18] Visser SN, Lesesne CA, Perou R (2007). National estimates and factors associated with medication treatment for childhood attention-deficit/hyperactivity disorder. Pediatrics.

[19] Storebø OJ (2018). Methylphenidate for attention deficit hyperactivity disorder (ADHD) in children and adolescents—assessment of adverse events in non-randomised studies. Cochrane Database Syst. Rev..

[20] Swanson J (2003). Compliance with stimulants for attention-deficit/hyperactivity disorder: issues and approaches for improvement. CNS Drugs.

[21] Pharmaceuticals and Medical Devices Agency. https://www.pmda.go.jp/PmdaSearch/iyakuDetail/ResultDataSetPDF/800155_1179009G1022_1_17. Accessed Jan 2021.

[22] Statistics_Bureau_of_Japan. *Japanese Population on the First Day of Every Month.* https://www.stat.go.jp/data/jinsui/2.html#monthly. Accessed Jan 2021.

[23] Ching C, Eslick GD, Poulton AS (2019). Evaluation of methylphenidate safety and maximum-dose titration rationale in attention-deficit/hyperactivity disorder: a meta-analysis. JAMA Pediatr..

[24] Razani N (2015). Neighborhood characteristics and ADHD: results of a national study. J. Atten. Disord..

[25] Lash TL (2009). Applying Quantitative Bias Analysis to Epidemiologic Data.

[26] Bachmann CJ (2017). Trends in ADHD medication use in children and adolescents in five western countries, 2005–2012. Eur. Neuropsychopharmacol. J. Eur. Coll. Neuropsychopharmacol..

[27] McCarthy S (2012). The epidemiology of pharmacologically treated attention deficit hyperactivity disorder (ADHD) in children, adolescents and adults in UK primary care. BMC Pediatr..

[28] Stephenson CP, Karanges E, McGregor IS (2013). Trends in the utilisation of psychotropic medications in Australia from 2000 to 2011. Aust. N. Zeal. J. Psychiatry.

[29] Zuvekas SH, Vitiello B (2012). Stimulant medication use in children: a 12-year perspective. Am. J. Psychiatry.

[30] Okumura Y (2019). Prevalence, incidence and persistence of ADHD drug use in Japan. Epidemiol. Psychiatr. Sci..

[31] Burcu M, Zito JM, Metcalfe L, Underwood H, Safer DJ (2016). Trends in stimulant medication use in commercially insured youths and adults, 2010–2014. JAMA Psychiatry.

[32] Karlstad Ø, Furu K, Stoltenberg C, Håberg SE, Bakken IJ (2017). ADHD treatment and diagnosis in relation to children's birth month: Nationwide cohort study from Norway. Scand. J. Public Health.

[33] Beau-Lejdstrom R, Douglas I, Evans SJW, Smeeth L (2016). Latest trends in ADHD drug prescribing patterns in children in the UK: prevalence, incidence and persistence. BMJ Open.

[34] Kovess V (2015). Psychotropic medication use in French children and adolescents. J. Child Adolesc. Psychopharmacol..

[35] Piovani D, Clavenna A, Cartabia M, Bonati M (2016). Psychotropic medicine prescriptions in Italian youths: a multiregional study. Eur. Child Adolesc. Psychiatry.

[36] Bachmann CJ, Philipsen A, Hoffmann F (2017). ADHD in Germany: trends in diagnosis and pharmacotherapy. Dtsch. Arztebl. Int..

[37] Bolea-Alamañac B (2014). Evidence-based guidelines for the pharmacological management of attention deficit hyperactivity disorder: update on recommendations from the British Association for Psychopharmacology. J. Psychopharmacol. (Oxford).

[38] Kooij SJJ (2010). European consensus statement on diagnosis and treatment of adult ADHD: the European Network Adult ADHD. BMC Psychiatry.

[39] National Institute for Health and Care Excellence. Attention deficit hyperactivity disorder: diagnosis and management. https://www.nice.org.uk/guidance/ng87 (2019). 29634174

[40] Cortese S (2018). Comparative efficacy and tolerability of medications for attention-deficit hyperactivity disorder in children, adolescents, and adults: a systematic review and network meta-analysis. Lancet Psychiatry.

[41] Liu Q, Zhang H, Fang Q, Qin L (2017). Comparative efficacy and safety of methylphenidate and atomoxetine for attention-deficit hyperactivity disorder in children and adolescents: meta-analysis based on head-to-head trials. J. Clin. Exp. Neuropsychol..

[42] Howland RH (2009). Medication holidays. J. Psychosoc. Nurs. Ment. Health Serv..

[43] Hodgkins P, Sasane R, Christensen L, Harley C, Liu F (2011). Treatment outcomes with methylphenidate formulations among patients with ADHD: retrospective claims analysis of a managed care population. Curr. Med. Res. Opin..

[44] Lachaine J, Beauchemin C, Sasane R, Hodgkins PS (2012). Treatment patterns, adherence, and persistence in ADHD: a Canadian perspective. Postgrad. Med..

[45] Marcus SC, Durkin M (2011). Stimulant adherence and academic performance in urban youth with attention-deficit/hyperactivity disorder. J. Am. Acad. Child Adolesc. Psychiatry.

[46] Rucklidge JJ (2010). Gender differences in attention-deficit/hyperactivity disorder. Psychiatr. Clin. N. Am..

[47] Cornforth C, Sonuga-Barke E, Coghill D (2010). Stimulant drug effects on attention deficit/hyperactivity disorder: a review of the effects of age and sex of patients. Curr. Pharm. Des..

[48] Katzman MA, Sternat T (2014). A review of OROS methylphenidate (Concerta(®)) in the treatment of attention-deficit/hyperactivity disorder. CNS Drugs.

[49] Storebø OJ (2015). Methylphenidate for attention-deficit/hyperactivity disorder in children and adolescents: cochrane systematic review with meta-analyses and trial sequential analyses of randomised clinical trials. BMJ.

[50] Treuer T (2013). A systematic review of combination therapy with stimulants and atomoxetine for attention-deficit/hyperactivity disorder, including patient characteristics, treatment strategies, effectiveness, and tolerability. J. Child Adolesc. Psychopharmacol..

[51] Hvolby A (2015). Associations of sleep disturbance with ADHD: implications for treatment. Atten. Defic. Hyperact. Disord..

[52] Martins R (2019). Sleep disturbance in children with attention-deficit hyperactivity disorder: a systematic review. Sleep Sci..

[53] Wajszilber D, Santiseban JA, Gruber R (2018). Sleep disorders in patients with ADHD: impact and management challenges. Nat. Sci. Sleep.

[54] Corkum P (2020). The effects of extended-release stimulant medication on sleep in children with ADHD. J. Can. Acad. Child Adolesc. Psychiatry.

[55] Kidwell KM, Van Dyk TR, Lundahl A, Nelson TD (2015). Stimulant medications and sleep for youth with ADHD: a meta-analysis. Pediatrics.

