# Supplementary material for: Risk factors for low adherence to methylphenidate treatment in pediatric patients with attention-deficit/hyperactivity disorder
Source: Sci Rep. 2021 Jan 18;11:1707. doi: 10.1038/s41598-021-81416-z (PMC7814069; doi:10.1038/s41598-021-81416-z)
Supplement: Supplementary file 1 — Supplementary Information. [file 41598_2021_81416_MOESM1_ESM.pdf]

Supplementary information for

Risk factors for low adherence to methylphenidate treatment in pediatric patients with attention-deficit/hyperactivity disorder

Asami Ishizuya[1,2,3], Minori Enomoto[4], Hisateru Tachimori[6], Hidehiko Takahashi[1], Genichi Sugihara[1], Shingo Kitamura[3], Kazuo Mishima[2,3,5]*

1 Department of Psychiatry and Behavioral Neurosciences, Graduate School of Medical and Dental Sciences, Tokyo Medical and Dental University, Bunkyo-ku, Tokyo, Japan

2 Department of Neuropsychiatry, Akita University Graduate School of Medicine, Akita, Japan

3 Department of Sleep-Wake Disorders, National Institute of Mental Health, National Center of Neurology and Psychiatry, Kodaira, Tokyo, Japan

4 Department of Medical Technology, School of Health Sciences, Tokyo University of Technology, Tokyo, Japan

5 International Institute for Integrative Sleep Medicine, University of Tsukuba, Tsukuba, Japan

6 Department of Clinical Epidemiology, Translational Medical Center, National Center of Neurology and Psychiatry, Kodaira, Tokyo, Japan

Supplementary Table S1. Sensitivity analysis.

**Effect of ADHD severity on the association between adherence and the mean daily dosage prescribed in the first 3 months**				**Effect of adverse events on the association between adherence and concomitant hypnotic use**			
Estimated RR of confounding factor	Prevalence		Adjusted RR	Estimated RR of confounding factor	Prevalence		Adjusted RR
	Mean daily dosage prescribed in the first 3 months				Concomitant hypnotics use		
	<18 mg/day	≥18 mg/day			With	Without	
1.6	50	70	1.68	1.5	60	50	1.46
	50	60	1.61		60	40	1.4
	40	70	1.76		70	60	1.46
	40	60	1.68		70	50	1.41
	30	70	1.85		70	40	1.35
	30	60	1.77		80	60	1.41
					80	50	1.36
2	50	70	1.74	2	60	50	1.42
	50	60	1.64		60	40	1.33
	40	70	1.86		70	60	1.43
	40	60	1.75		70	50	1.34
	30	70	2.01		70	40	1.25
	30	60	1.89		80	60	1.35
					80	50	1.27
3	50	70	1.84	3	60	50	1.38
	50	60	1.69		60	40	1,24
	40	70	2.05		70	60	1.39
	40	60	1.88		70	50	1.27
	30	70	2.3		70	40	1.14
	30	60	2.11		80	60	1.28
					80	50	1.17

RR, risk ratio.

Supplementary Table S2. List of psychotropics included in this study.

**Antipsychotics**					
ID	Generic name	ID	Generic name	ID	Generic name
M01	bromperidol	M14	olanzapine	M27	timiperone
M02	carpipramine	M15	oxypertine	M28	trifluoperazine
M03	chlorpromazine	M16	perospirone	M29	zotepine
M04	clocapramine	M17	perphenazine	M30	tiapride
M05	floropipamide	M18	pimozide	M31	blonanserin
M06	fluphenazine	M19	prochlorperazine	M32	aripiprazole
M07	fluphenazine decanoate	M20	propericyazine	M33	clozapine
M08	haloperidol	M21	quetiapine	M34	paliperidone
M09	haloperidol decanoate	M22	risperidone	M35	risperidone
M10	levomepromazine	M23	spiperone	M36	chlorpromazine + promethazine + phenobarbital
M11	moperone	M24	sulpiride	M37	asenapine
M12	mosapramine	M25	sultopride	M38	brexpiprazole
M13	nemonapride	M26	thioridazine		
**Antidepressants**					
ID	Generic name	ID	Generic name	ID	Generic name
D01	amitriptyline	D09	maprotiline	D17	sulpiride
D02	amoxapine	D10	mianserin	D18	trazodone
D03	clomipramine	D11	milnacipran	D19	trimipramine
D04	desipramine	D12	nortriptyline	D20	duloxetine
D05	dosulepine	D13	paroxetine	D21	mirtazapine
D06	fluvoxamine	D14	safrazine	D22	escitalopram
D07	imipramine	D15	sertraline	D23	venlafaxine hydrochloride
D08	lofepramine	D16	setiptiline		
**Anxiolytics**					
ID	Generic name	ID	Generic name	ID	Generic name
A01	alprazolam	A08	etizolam	A15	medazepam
A02	bromazepam	A09	fludiazepam	A16	mexazolam
A03	chlordiazepoxide	A10	flutazolam	A17	oxazepam
A04	clorazepate	A11	flutoprazepam	A18	oxazolam
A05	clotiazepam	A12	hydroxyzine	A19	prazepam
A06	cloxazolam	A13	loflazepate	A20	tandospirone
A07	diazepam	A14	lorazepam	A21	tofisopam
**Hypnotics**					
ID	Generic name	ID	Generic name	ID	Generic name

S01	amobarbital	S11	flurazepam	S21	rilmazafone
S02	barbital	S12	haloxazolam	S22	secobarbital
S03	bromovalerylurea	S13	lormetazepam	S23	triazolam
S04	brotizolam	S14	midazolam	S24	triclofos sodium
S05	butoctamide	S15	nimetazepam	S25	zolpidem
S06	chloral hydrate	S16	nitrazepam	S26	zopiclone
S07	clonazepam	S17	passiflora extract	S27	ramelteon
S08	estazolam	S18	pentobarbital	S28	eszopiclone
S09	etizolam	S19	phenobarbital	S29	suvorexant
S10	flunitrazepam	S20	quazepam		
**Antiepileptics**					
ID	Generic name	ID	Generic name	ID	Generic name
E01	phenobarbital	E11	trimethadione	E21	phenytoin + phenobarbital + sodium benzoate
E02	clonazepam	E12	metarbital	E22	fosphenytoin sodium hydrate
E03	phenytoin	E13	ethotoin	E23	stiripentol
E04	carbamazepine	E14	sultiame	E24	rufinamide
E05	sodium valproate	E15	acetyl pheneturide	E25	perampanel hydrate
E06	ethosuximide	E16	gabapentin	E26	oxcarbazepine
E07	zonisamide	E17	topiramate	E27	vigabatrin
E08	acetazolamide sodium	E18	lamotrigine	E28	lacosamide
E09	clobazam	E19	levetiracetam		
E10	primidone	E20	phenytoin + phenobarbital		
**ADHD medications**					
ID	Generic name	ID	Generic name	ID	Generic name
Z02	methylphenidate hydrochloride	Z09	atomoxetine hydrochloride		
